# Exosomal Phosphorylated IRS1 and Age-Dependent Risk for Depression and Bipolar Disorder in the Netherlands Study of Depression and Anxiety

**DOI:** 10.1192/j.eurpsy.2026.10926

**Published:** 2026-07-31

**Authors:** K. T. Watson, N. Rasgon, A. Evers, M. Bot, E. Eitan, K. Haque, A. F. Schatzberg, B. W. Penninx

**Affiliations:** 1 Psychiatry & Behavioral Sciences, Stanford University, Stanford; 2 Psychiatry, Icahn School of Medicine at Mount Sinai, New York, United States; 3 Psychiatry, Vrije Universiteit Amsterdam, Amsterdam, Netherlands; 4 NeuroDex, Natick; 5 Psychiatry, Vrije Universiteit Amsterdam, Amsterdam, United States

## Abstract

**Introduction:**

Exosomes offer a unique window into central insulin signaling through vesicles that can be isolated from peripheral blood. While peripheral insulin resistance has been associated with mood and metabolic disorders, the contribution of neuronal insulin signaling to affective psychopathology is less well understood. Clarifying whether insulin-related abnormalities in the brain contribute to depression and bipolar disorder, particularly across the lifespan, may reveal mechanisms of persistence and recurrence not explained by peripheral measures alone.

**Objectives:**

This study aimed to determine whether levels of phosphorylated IRS1 (pIRS1) in neuron-derived exosomes are associated with psychiatric diagnostic status in the Netherlands Study of Depression and Anxiety (NESDA). A further objective was to assess whether such associations vary as a function of age and whether they remain independent of peripheral metabolic markers.

**Methods:**

Data were obtained from NESDA, a large, well-characterized psychiatric epidemiology cohort. Diagnostic groups included healthy controls, individuals with current major depressive disorder (MDD), remitted MDD, anxiety without MDD, and bipolar disorder. Multinomial logistic regression models evaluated associations between exosomal pIRS1 and diagnosis, adjusting for batch effects, age, sex, education, and metabolic indices including waist circumference, triglycerides, BMI, and fasting insulin.

**Results:**

Individuals with MDD or bipolar disorder were older, more often female, and displayed less favorable metabolic profiles relative to controls. Overall, pIRS1 was not associated with diagnosis. However, a significant interaction with age showed that higher pIRS1 was linked to increased odds of current MDD, remitted MDD, and bipolar disorder compared with controls, whereas no such association was observed for anxiety disorders. Importantly, these associations persisted after controlling for peripheral metabolic measures, suggesting that the observed effects were not reducible to peripheral insulin resistance.

**Conclusions:**

The findings indicate that central insulin signaling abnormalities, as reflected in exosomal pIRS1, may underlie an age-dependent vulnerability to depression and bipolar disorder. By capturing aspects of insulin dysregulation that peripheral measures cannot, exosomal biomarkers provide novel insight into the biological persistence of affective disorders and may help identify subgroups of patients at heightened risk for recurrence or chronicity.

**Disclosure of Interest:**

None Declared

